# Supplementary material for: A clinical trial to evaluate the dayzz smartphone app on employee sleep, health, and productivity at a large US employer
Source: PLoS One. 2022 Jan 5;17(1):e0260828. doi: 10.1371/journal.pone.0260828 (PMC8730427; doi:10.1371/journal.pone.0260828)
Supplement: S1 File — (DOCX) [file pone.0260828.s001.docx]

Answer all questions accurately and completely in order to provide the PHRC with the relevant information to assess the risk-benefit ratio for the study. Do not leave sections blank.

# PRINCIPAL/OVERALL INVESTIGATOR

Laura K. Barger, Ph.D.

# PROTOCOL TITLE

Operational trial of Dayzz app

# FUNDING

Dayzz Live Well Ltd.

# VERSION DATE

January 12, 2021

### SPECIFIC AIMS

Concisely state the objectives of the study and the hypothesis being tested.

We have shown that sleep health education and sleep disorders screening improve health and safety of employees. We hypothesize that the Dayzz app, offering a personalized, scientifically based sleep training plan, might further promote improved health, safety and well-being. Therefore, we will conduct a clinical trial of the sleep health education and sleep disorders screening program in combination with the Dayzz app.

# BACKGROUND AND SIGNIFICANCE

Provide a brief paragraph summarizing prior experience important for understanding the proposed study and procedures.

Sleep deficiency is a hidden cost of our tech-driven 24-7 society, with 70% of Americans admitting that they routinely obtain insufficient sleep. The issue is pervasive in our workforce, as 30% of US workers report sleeping less than 6 hours per night. Additionally, it is estimated that 50-70 million individuals have a sleep disorder. Undiagnosed and untreated sleep disorders are associated with poor health outcomes for the individual and generate substantial costs for the employer. Diminished alertness resulting from sleep deficiency or undiagnosed and untreated sleep disorders contributes to absenteeism (hours of work missed), presenteeism (diminished work performance at work), health care expenditures, occupational injuries, workplace accidents, and commute-related motor vehicle crashes; all of which result in substantial direct and indirect costs. We have shown that adverse impacts on employees and employers can be mitigated through a sleep health education and sleep disorder screening program which we have implemented in a variety of industries. Further, healthcare apps are becoming increasing popular. Dayzz has created a personalized sleep training app that evaluates sleep issues and offers a complete, holistic sleep improvement solution. It is unique in that the sleep app uses evidence-based, big-data-based algorithms to provide personalized sleep training plans. The sleep solutions are tailored to the individual user’s lifestyle and sleep needs. Using unique motivational strategies and continuous supportive messaging, Dayzz encourages individuals to reach their wellness goals. User’s behavioral changes are rewarded through feedback and encouragement. The Dayzz app learns about participant’s daily habits and sleep schedules based on what information is entered in to the app. The Dayzz algorithm will then determine a personalized sleep improvement plan. The Dayzz app provides feedback such as daily reminders, tips and customized challenges to the participant to continually improve their sleep. For example, the participant could be challenged with quick tasks, e.g. not drinking caffeine in the afternoon, for a whole week. The app tracks the participant’s progress and adjusts the plan with new tasks based on performance. The training plan is reflected on a dashboard, where the participant earns trophies for successfully completing tasks, such as caffeine consumption, exposure to light at appropriate times, or adjusting to a new bedtime. Participants are engaged through timed reminders and motivation boosts based on their location, motion, and activity. Examples include sending the participant a reminder to drink his/her last cup of coffee when recognizing a restaurant presence or calculating his/her total daylight exposure time based on time spent outdoors. There are four education training modules that are available in the app: Days (caffeine use, light exposure), Nights (behaviors affecting sleep quantity and quality, Environment (room temperature, noise), and Mind (sleep-related anxieties, meditation). We hypothesize that the Dayzz app, offering a personalized, scientifically based sleep training plan, might further promote improved health, safety and well-being. We will conduct a clinical trial of the sleep health education and sleep disorders screening program in combination with the Dayzz app.

###### RESEARCH DESIGN AND METHODS

Briefly describe study design and anticipated enrollment, i.e., number of subjects to be enrolled by researchers study-wide and by Partners researchers. Provide a brief summary of the eligibility criteria (for example, age range, gender, medical condition). Include any local site restrictions, for example, “Enrollment at Partners will be limited to adults although the sponsor’s protocol is open to both children and adults.”

We will conduct a single-site, randomized, controlled trial using a wait-list control design. We plan to enroll 2000 daytime employees into the study, 1000 randomly assigned to the experimental Early Dayzz group and 1000 to the control Later Dayzz group.

Eligible participants will be daytime employees. Potential subjects will be directed to a website landing page with more information about the study. If interested, the potential subject is screened for study eligibility and if eligible presented with the online consent form. Subjects who consent to participate will then be randomized into the intervention (Early Dayzz) or control (Later Dayzz) groups.

Eligible participants must own and use a smart phone, nominally work a day schedule, and regularly use an app (i.e., once per week). Potential participants will be excluded if they are regularly working evening, night or rotating shifts or are pregnant or breast-feeding.

Briefly describe study procedures. Include any local site restrictions, for example, “Subjects enrolled at Partners will not participate in the pharmacokinetic portion of the study.” Describe study endpoints.

After consenting, participants randomized to the Early Dayzz group will take a short online Initial Contact questionnaire covering basic demographics. They will then receive a 20-minute online Sleep Health and Wellness (SHAW) presentation. Immediately after the presentation the subject will take a short Sleep Disorders Risk Assessment and be shown the results. Finally, the Early Dayzz subjects will be invited to download the Dayzz app and register using their email address. They will be encouraged to use the Dayzz app throughout the up to 9-month study interval and if they use a wearable device (e.g., Fitbit), they can choose to link the wearable to the Dayzz app to receive further feedback.

Participants in Later Dayzz control condition will be able to view a short presentation about the study and the importance of controls in randomized trials. Participants will receive the SHAW program and access to the Dayzz app at the end of the study period.

All participants (experimental and control) will receive an email with a link to the baseline questionnaire and the first monthly questionnaire. For up to 9 months following, on the 28th of each month, each participant will receive an email with a link to the monthly questionnaire. At the end of the study period, each participant will receive a link to complete the end-of-study questionnaire. Each participant will also complete a daily eDiary for one week within the ~first month of enrollment and for one week within the ~third month of the study. All questionnaires and diaries are completed online.

For studies involving treatment or diagnosis, provide information about standard of care at Partners (e.g., BWH, MGH) and indicate how the study procedures differ from standard care. Provide information on available alternative treatments, procedures, or methods of diagnosis.

This study does not provide diagnosis or treatment, per se. Experimental participants will be provided the SHAW program at the outset of the study, including screening for common sleep disorders. Those who screen at risk for OSA or RLS will be encouraged to make an appointment at a sleep disorders clinic. Those who screen at risk for insomnia will be given the opportunity to use the Dayzz app insomnia behavioral therapy program. The control group will be given the sleep health and wellness education program, sleep disorders screening, appointment options and Dayzz app at the end of the nine months of data collection.

There are no alternative treatments or procedures, other than the subject exercising his or her right not to participate in the study.

Describe how risks to subjects are minimized, for example, by using procedures which are consistent with sound research design and which do not unnecessarily expose subjects to risk or by using procedures already being performed on the subject for diagnostic or treatment purposes.

Sound research design procedures are used in this study. There are minimal risks to participating in the Dayzz study. Participants are asked to answer questions on surveys and as part of a daily diary. Participants may experience some psychological discomfort responding to personal questions such as those about sleep, health and safety outcomes. Participants may choose not to answer any questions that they are not comfortable answering. We will protect the confidentiality of the participants and their data by segregating identifiable information from survey responses.

Describe explicitly the methods for ensuring the safety of subjects. Provide objective criteria for removing a subject from the study, for example, objective criteria for worsening disease/lack of improvement and/or unacceptable adverse events. The inclusion of objective drop criteria is especially important in studies designed with placebo control groups.

This is a study with minimal risk. Participants complete online questionnaires and diaries. There are no objective criteria for removing a subject from the study. A participant may drop out at any time if he or she wishes to do so.

# FORESEEABLE RISKS AND DISCOMFORTS

Provide a brief description of any foreseeable risks and discomforts to subjects. Include those related to drugs/devices/procedures being studied and/or administered/performed solely for research purposes. In addition, include psychosocial risks, and risks related to privacy and confidentiality. When applicable, describe risks to a developing fetus or nursing infant.

We believe there are no risks or discomforts associated with completing the web-based questionnaires or completing the sleep-work diary. There is a very small risk inherent in any type of data collection that confidentiality may be compromised. We will make great efforts to ensure the confidentiality of the information. The investigators reserve the right to terminate the study at any time they feel it is necessary for the subject's welfare, or for research purposes.

**EXPECTED BENEFITS**

# Describe both the expected benefits to individual subjects participating in the research and the importance of the knowledge that may reasonably be expected to result from the study. Provide a brief, realistic summary of potential benefits to subjects, for example, “It is hoped that the treatment will result in a partial reduction in tumor size in at least 25% of the enrolled subjects.” Indicate how the results of the study will benefit future patients with the disease/condition being studied and/or society, e.g., through increased knowledge of human physiology or behavior, improved safety, or technological advances.

We do not expect there to be any direct benefits resulting from participation in the questionnaires or diaries. Those who use the Dayzz app may learn information about their sleep patterns and the tips Dayzz provides on healthy sleep practices may lead to improved sleep in some participants.

Approximately 70% of Americans report that they routinely get insufficient sleep. The issue is pervasive in the workforce, with 30% of U.S. workers and 44% of night shift workers reporting less than six hours of sleep on average. If our study successfully shows that the Dayzz app is effective in improving sleep, health and well-being of employees, it may be widely deployed.

**EQUITABLE SELECTION OF SUBJECTS**

The risks and benefits of the research must be fairly distributed among the populations that stand to benefit from it. No group of persons, for example, men, women, pregnant women, children, and minorities, should be categorically excluded from the research without a good scientific or ethical reason to do so. Please provide the basis for concluding that the study population is representative of the population that stands to potentially benefit from this research.

We intend to study employees who work day shifts. The Dayzz app was designed to improve the sleep of such an employee group. Children are not included as the Dayzz app was developed for adults. Men, women and minorities are all included. Pregnant and breast-feeding mothers are excluded as recommendations on sleep may not be appropriate for them.

When people who do not speak English are excluded from participation in the research, provide the scientific rationale for doing so. Individuals who do not speak English should not be denied participation in research simply because it is inconvenient to translate the consent form in different languages and to have an interpreter present.

Enrollment of participants who do not understand English is limited only to the extent that the study population contains these groups.

For guidance, refer to the following Partners policy:

Obtaining and Documenting Informed Consent of Subjects who do not Speak English

[**https://www.partners.org/Assets/Documents/Medical-Research/Clinical-Research/Non-English-Speaking-Subjects.pdf**](https://www.partners.org/Assets/Documents/Medical-Research/Clinical-Research/Non-English-Speaking-Subjects.pdf)

**RECRUITMENT PROCEDURES**

Explain in detail the specific methodology that will be used to recruit subjects. Specifically address how, when, where and by whom subjects will be identified and approached about participation. Include any specific recruitment methods used to enhance recruitment of women and minorities.

Daytime employees will be recruited for this study. Potential subjects will be directed to a website landing page with more information about the study via IRB-approved recruitment platforms (e.g., Rally) and email messaging from institutional stakeholders or study PI.

There are no specific recruitment procedures to enhance the recruitment of women or minorities.

Provide details of remuneration, when applicable. Even when subjects may derive medical benefit from participation, it is often the case that extra hospital visits, meals at the hospital, parking fees or other inconveniences will result in additional out-of-pocket expenses related to study participation. Investigators may wish to consider providing reimbursement for such expenses when funding is available

Participants who complete the full enrollment procedure (e.g., for Early Dayzz: Initial Contact, SHAW, Sleep Disorders Risk Assessment, download Dayzz app) will receive a $25 gift card. After completing the first round of eDiaries, participants can receive a sleep shirt ($10 value). Participants who complete the monthly survey will be eligible for that month’s drawing with two subjects winning Air Pods Pro ($250). For completing the December 2020 survey, participants will be eligible to win 1 of 10 AirPods Pro (increased from two to help boost participation and engagement). Each monthly survey completed and each week of eDiaries completed will earn the participant chances in the end-of-study random prize drawing totaling $10,000: 10 $500 prizes, 10 $250 prizes and 25 $100 prizes.

For guidance, refer to the following Partners policies:

Recruitment of Research Subjects

[**https://www.partners.org/Assets/Documents/Medical-Research/Clinical-Research/Recruitment-Of-Research-Subjects.pdf**](https://www.partners.org/Assets/Documents/Medical-Research/Clinical-Research/Recruitment-Of-Research-Subjects.pdf)

Guidelines for Advertisements for Recruiting Subjects

[**https://www.partners.org/Assets/Documents/Medical-Research/Clinical-Research/Guidelines-for-Advertisements.pdf**](https://www.partners.org/Assets/Documents/Medical-Research/Clinical-Research/Guidelines-for-Advertisements.pdf)

Remuneration for Research Subjects

[**https://www.partners.org/Assets/Documents/Medical-Research/Clinical-Research/Remuneration-for-Research-Subjects.pdf**](https://www.partners.org/Assets/Documents/Medical-Research/Clinical-Research/Remuneration-for-Research-Subjects.pdf)

#### CONSENT PROCEDURES

Explain in detail how, when, where, and by whom consent is obtained, and the timing of consent (i.e., how long subjects will be given to consider participation). For most studies involving more than minimal risk and all studies involving investigational drugs/devices, a licensed physician investigator must obtain informed consent. When subjects are to be enrolled from among the investigators’ own patients, describe how the potential for coercion will be avoided.

Potential subjects will be directed to a website landing page with more information about the study. If interested, the potential subject is screened for study eligibility and if eligible presented with the online consent form. There are no limits on how long subjects will be given to consider participation. Potential subjects may take as long as they need to review the consent form and leave and return to consent as many times as needed.

NOTE: When subjects are unable to give consent due to age (minors) or impaired decision-making capacity, complete the forms for Research Involving Children as Subjects of Research and/or Research Involving Individuals with Impaired Decision-making Capacity, available on the New Submissions page on the PHRC website:

[**https://partnershealthcare.sharepoint.com/sites/phrmApply/aieipa/irb**](https://partnershealthcare.sharepoint.com/sites/phrmApply/aieipa/irb)

For guidance, refer to the following Partners policy:

Informed Consent of Research Subjects:

[**https://www.partners.org/Assets/Documents/Medical-Research/Clinical-Research/Informed-Consent-of-Research-Subjects.pdf**](https://www.partners.org/Assets/Documents/Medical-Research/Clinical-Research/Informed-Consent-of-Research-Subjects.pdf)

## DATA AND SAFETY MONITORING

Describe the plan for monitoring the data to ensure the safety of subjects. The plan should include a brief description of (1) the safety and/or efficacy data that will be reviewed; (2) the planned frequency of review; and (3) who will be responsible for this review and for determining whether the research should be altered or stopped. Include a brief description of any stopping rules for the study, when appropriate. Depending upon the risk, size and complexity of the study, the investigator, an expert group, an independent Data and Safety Monitoring Board (DSMB) or others might be assigned primary responsibility for this monitoring activity.

NOTE: Regardless of data and safety monitoring plans by the sponsor or others, the principal investigator is ultimately responsible for protecting the rights, safety, and welfare of subjects under his/her care.

A data safety monitoring board is not required for this low risk survey study. The PI, with assistance from the study personnel, will be responsible for safety monitoring, by reviewing data on a regular basis (e.g., once per month). Survey data on sleep duration and well-being and free text comments will be reviewed.

Describe the plan to be followed by the Principal Investigator/study staff for review of adverse events experienced by subjects under his/her care, and when applicable, for review of sponsor safety reports and DSMB reports. Describe the plan for reporting adverse events to the sponsor and the Partners’ IRB and, when applicable, for submitting sponsor safety reports and DSMB reports to the Partners’ IRBs. When the investigator is also the sponsor of the IND/IDE, include the plan for reporting of adverse events to the FDA and, when applicable, to investigators at other sites.

NOTE: In addition to the adverse event reporting requirements of the sponsor, the principal investigator must follow the Partners Human Research Committee guidelines for Adverse Event Reporting

The PI will be ultimately responsible for reviewing and reporting all adverse events and unanticipated problems to the IRB, as required. She will review the data and meet with the study team monthly to discuss any problems (anticipated or unanticipated) with the research study, as well as any adverse events. In addition to adverse events, the PI and study staff will regularly review study progress to identify unanticipated problems, and report these to the IRB, as required.

## MONITORING AND QUALITY ASSURANCE

Describe the plan to be followed by the principal investigator/study staff to monitor and assure the validity and integrity of the data and adherence to the IRB-approved protocol. Specify who will be responsible for monitoring, and the planned frequency of monitoring. For example, specify who will review the accuracy and completeness of case report form entries, source documents, and informed consent.

NOTE: Regardless of monitoring plans by the sponsor or others, the principal investigator is ultimately responsible for ensuring that the study is conducted at his/her investigative site in accordance with the IRB-approved protocol, and applicable regulations and requirements of the IRB.

Ongoing study progress, including enrollment, data quality/integrity, completeness, and safety issues, will be reviewed monthly by the study team. During these meetings the Principal Investigator and other members of the research team are present. eDiary entries will be checked daily by a study team member to ensure the participant is completing them and to follow-up in cases where there are missing or potentially incorrect entries.

For guidance, refer to the following Partners policies:

##### Data and Safety Monitoring Plans and Quality Assurance

[**https://www.partners.org/Assets/Documents/Medical-Research/Clinical-Research/DSMP-in-Human-Subjects-Research.pdf**](https://www.partners.org/Assets/Documents/Medical-Research/Clinical-Research/DSMP-in-Human-Subjects-Research.pdf)

Reporting Unanticipated Problems (including Adverse Events)

[**https://www.partners.org/Assets/Documents/Medical-Research/Clinical-Research/Reporting-Unanticipated-Problems-including-Adverse-Events.pdf**](https://www.partners.org/Assets/Documents/Medical-Research/Clinical-Research/Reporting-Unanticipated-Problems-including-Adverse-Events.pdf)

# PRIVACY AND CONFIDENTIALITY

Describe methods used to protect the privacy of subjects and maintain confidentiality of data collected. This typically includes such practices as substituting codes for names and/or medical record numbers; removing face sheets or other identifiers from completed surveys/questionnaires; proper disposal of printed computer data; limited access to study data; use of password-protected computer databases; training for research staff on the importance of confidentiality of data, and storing research records in a secure location.

NOTE: Additional measures, such as obtaining a Certificate of Confidentiality, should be considered and are strongly encouraged when the research involves the collection of sensitive data, such as sexual, criminal or illegal behaviors.

To maintain confidentiality of information obtained from the research participants, the computer systems containing confidential data will have a level and scope of security that equals or exceeds those required by HIPAA guidelines. The data is collected and stored centrally and securely at the institution and under the auspices of the institutional IT security team. Through our prior research, we have extensive experience with maintaining this level of data security. Only IRB approved investigators and study staff will have access to these identified data. Data from individual participants will be assigned alpha-numerical identification codes, which will be used to link data records for each individual participant. This ensures that the study data will be de-identified before internal sharing and analysis within the research team. The PHI-code index will be maintained in a separate secure location, encrypted file and on a password-protected, IP-restricted computer system. We understand our responsibilities to protect the confidentiality of research and statistical information and have developed specific procedures to ensure that this information is only used or revealed in accordance with its intended research purposes.

SENDING SPECIMENS/DATA TO RESEARCH COLLABORATORS OUTSIDE PARTNERS

Specimens or data collected by Partners investigators will be sent to research collaborators outside Partners, indicate to whom specimens/data will be sent, what information will be sent, and whether the specimens/data will contain identifiers that could be used by the outside collaborators to link the specimens/data to individual subjects.

Participants will download the Dayzz app and register using their cell phone number or email address. Dayzz will provide to us engagement data (e.g., number of tasks completed, days and times of use of app) based on the cell phone or email address via the encrypted Partners Secure File Transfer (transfer.partners.org). We will link with our other study data. Dayzz will not have access to and we will not share other study data with Dayzz.

Specifically address whether specimens/data will be stored at collaborating sites outside Partners for future use not described in the protocol. Include whether subjects can withdraw their specimens/data, and how they would do so. When appropriate, submit documentation of IRB approval from the recipient institution.

Only Dayzz app engagement data (e.g., number of tasks completed, days and times of use of app) are stored with Dayzz. These data are routinely collected by the Dayzz app and explained in the usage agreement when the app is downloaded.

Subjects can withdraw from the study and withdraw their data by sending an email to the investigators stating their intention to do so. If a participant wanted to withdraw any data that Dayzz had collected on their usage of the app, they could email Dayzz requesting that, and investigators would help facilitate that request.

# RECEIVING SPECIMENS/DATA FROM RESEARCH COLLABORATORS OUTSIDE PARTNERS

When specimens or data collected by research collaborators outside Partners will be sent to Partners investigators, indicate from where the specimens/data will be obtained and whether the specimens/data will contain identifiers that could be used by Partners investigators to link the specimens/data to individual subjects. When appropriate, submit documentation of IRB approval and a copy of the IRB-approved consent form from the institution where the specimens/data were collected.

Participants will download the Dayzz app and register using their cell phone or email address. Dayzz will provide to us engagement data (e.g., number of tasks completed, days and times of use of app) based on the cell phone or email address via the encrypted Partners Secure File Transfer (transfer.partners.org). We will link with our other study data using the cell phone number or email address. Those identifiers will then be stripped out of the database after linkage.
